# Subclavian central venous catheters guidance and examination by ultrasound: a randomized controlled study versus landmark method

**DOI:** 10.1186/2197-425X-3-S1-A603

**Published:** 2015-10-01

**Authors:** S Perbet, F Grimaldi, B Pereira, JM Constantin

**Affiliations:** CHU Clermont-Ferrand, CHU Estaing, Clermont-Ferrand, France; Réanimatin Adultes, CHU Clermont Ferrand, Clermont-Ferrand, France; Biostatistics Unit, CHU Clermont Ferrand, Clermont-Ferrand, France; Réanimation Adultes, CHU Clermont Ferrand, Clermont-Ferrand, France

## Introduction

Catheterization under ultrasound (US) guidance of the central venous catheters is now a part of recommendations. It was already validated for the jugular way, allowing to decrease the failures, to obtain a faster venous access and to reduce the complications. The US can allow also the check of the good position and the absence of complications.

## Objectives

The objective of the study SUBGEUS was to compare an “all” US technique (catheterization and control) versus the standard technique (cutaneous landmark and radiographic location), to estimate the times of subclavian catheters catheterization and their check.

## Methods

This single center, randomized, controlled study was approved by the Ethics Committee of Clermont-Ferrand and had included 300 patients requiring the catheterization of a central subclavian catheter and randomized into two groups: "all" US or standard (STD). The primary endpoint was the time between the start of the process until verification of the catheter. The secondary endpoints were: time of the different stages of the procedure, the occurrence of complications (pneumothorax, hematoma, arterial puncture), malpositions, the failure rate (> 2 punctures) and the number of punctures.

Different times were evaluated: T0 = disinfection, T1 = puncture, T2 = return of blood, T3 = dressing,

T4 = realized checking, T5 = correction made if necessary.

## Results

150 catheters were placed in each group. Age, BMI, male / female ratio, SOFA, IGS II, side of placement were not different. US has identified 10 cases of thrombosis. The median total time of T0-T4 procedure was shorter in the group US (1064 [840-1350] sec vs 1345 [1065.1730] sec, p = 0.0001). The time (in seconds) were US / STD: T0-T1 (310 [213-400] vs. 205 [138.255], p = 0.0001), T1-T2 (100 [55-222] vs 67 [25-145], p = 0.0005), T2-T3 (310 [245-394] vs. 278 [194-373], p = 0.01), T3-T4 (292 [190-397] vs. 672 [510-987], p = 0.001). The failure rate was not significantly different between the two groups (US = 16% vs STD = 19%, p = 0.54). There was no significant difference between the number of punctures (US 1.6 ± 1.5 vs STD 1.8 ± 1.6, p = 0.28), and malpositions (US = 20 vs STD = 22, p = 0.74). No pneumothorax was found, 8 arterial punctures (5 US vs 3 STD, p = 0.72), 13 hematomas (8 US vs 5 STD, p = 0.57). For 11 cases, chest radiography allowed to objectify undetected malposition by US.

## Conclusions

The first analysis of this RCT showed a decrease in total procedure time (catheterization + control) with a longer time of catheterization but a quick time of check for catheterization of subclavian venous catheters with US. The failure rate and the number of malpositions were not different. However, research by malposition US can be made difficult by poor echogenicity compared to the right atrium, the difficulty of identifying a flow turgid when tested contrast and malposition in venous confluence. Ultrasonography detected thrombosis in 7% of cases.

